# Global database of cement production assets and upstream suppliers

**DOI:** 10.1038/s41597-023-02599-w

**Published:** 2023-10-13

**Authors:** Nataliya Tkachenko, Kevin Tang, Matthew McCarten, Steven Reece, David Kampmann, Conor Hickey, Maral Bayaraa, Peter Foster, Courtney Layman, Cristian Rossi, Kimberly Scott, Dave Yoken, Christophe Christiaen, Ben Caldecott

**Affiliations:** 1 Smith School of Enterprise and the Environment, University of Oxford, South Parks Road, Oxford, OX1 3QY UK; 2 Lloyds Banking Group, Gresham Street, City of London, EC2V 7HN UK; 3 Judge Business School, University of Cambridge, Trumpington Street, Cambridge, CB2 1AG UK; 4 The Alan Turing Institute, Euston Road, London, NW1 2DB UK; 5 UK Centre for Greening Finance and Investment, University of Oxford, South Parks Road, Oxford, OX1 3QY UK; 6 University of Edinburgh Business School, University of Edinburgh, Buccleuch Place, Edinburgh, EH8 9JS UK; 7 Environmental Change Institute, University of Oxford, South Parks Road, Oxford, OX1 3QY UK; 8 Harvard Business School, Boston, MA 02163 USA; 9 Satellite Applications Catapult, Fermi Avenue, Didcot, OX11 0QR UK; 10 Department of Engineering Science, University of Oxford, Parks Road, Oxford, OX1 3PJ UK; 11 Astraea Earth, Monticello Avenue, Charlottesville, Virginia USA

**Keywords:** Environmental impact, Geography

## Abstract

Cement producers and their investors are navigating evolving risks and opportunities as the sector’s climate and sustainability implications become more prominent. While many companies now disclose greenhouse gas emissions, the majority from carbon-intensive industries appear to delegate emissions to less efficient suppliers. Recognizing this, we underscore the necessity for a globally consolidated asset-level dataset, which acknowledges production inputs provenance. Our approach not only consolidates data from established sources like development banks and governments but innovatively integrates the age of plants and the sourcing patterns of raw materials as two foundational variables of the asset-level data. These variables are instrumental in modeling cement production utilization rates, which in turn, critically influence a company’s greenhouse emissions. Our method successfully combines geospatial computer vision and Large Language Modelling techniques to ensure a comprehensive and holistic understanding of global cement production dynamics.

## Background & Summary

The cement industry plays a crucial role in the global economy as it is used in the production of various infrastructure and construction projects. At the same time, global cement industry is one of the largest producers of carbon dioxide emissions, contributing to over 7% of the world’s total GHG budget. The production process of cement releases large amounts of CO2, mainly due to the high-temperature firing of raw materials, such as limestone and clay, but also due to energy- and resource- inefficient production technologies. This has a significant impact on the environment and contributes to global climate change^1–5^. Despite this, the cement industry continues to grow, driven by increasing demand for infrastructure development in emerging economies and urbanization in developed countries. According to the World Cement Association, global cement production is expected to reach 8.2 billion tonnes by 2030^6–9^.

Thus, in Western Europe^9,10^, the cement industry has seen a steady growth in demand, with major players such as HeidelbergCement, LafargeHolcim, and CRH making significant investments in their production facilities. In this region the cement industry is well established and has been facing challenges from stricter environmental regulations and declining demand due to the region’s sluggish economic growth. In North America^11,12^, the cement industry has been facing challenges due to the recently implemented trade policies and tariffs, which have affected the cost of raw materials for cement production. Despite this, the industry has seen growth in recent years, particularly in the United States, driven by the growth of the housing and construction markets, as well as by the shale gas boom and increased investment in infrastructure. In South America, the cement industry is dominated by a few major players such as CEMEX and Holcim, and the industry has seen growth driven by the increasing demand for infrastructure and construction projects, particularly in Brazil and Argentina. In Russia and the ex-Soviet states, until recently the cement industry has been growing due to the governments’ focus on improving regional infrastructures. Major players in the region include Eurocement and Heidelberg Materials, who are investing in their production facilities to meet the growing demand for development and update of the existing transport infrastructure and housing requirements. However, the economic sanctions imposed on Russia and the fall in oil prices have affected the industry’s growth in the region. Africa, which has a large population and an emerging middle class, is a growing market for the cement industry, but is also facing challenges such as a lack of infrastructure and political instability in some countries. Similarly to other regions, the cement industry has seen significant leap in recent years, driven by large infrastructure and construction projects. Major players in the region include Dangote Cement and LafargeHolcim. Australia and Asia are among the fastest-growing regions for the cement industry, with China being the largest producer and consumer of cement in the world^13–15^. However, the industry’s growth in Asia is also accompanied by environmental concerns, such as air pollution and deforestation. In Australia, the cement industry has seen slow growth in recent years, with major players remaining Adelaide Brighton and Boral Cement. In Asia, the cement industry is dominated by a few major players such as China National Building Materials (CNBM) and Anhui Conch, who have been expanding their operations in the region. The industry has seen significant growth driven by the increasing demand for infrastructure/construction projects, particularly in China, India, and Southeast Asia.

Obtaining reliable data sources on individual cement production facilities is a major challenge for the industry^16–21^. This is due to the lack of transparency and standardized reporting practices, as well as the complexity of the production process. Data scientists are now using new methods, such as satellite imagery and machine learning, to better understand the industry’s impact on the environment and track production activities. These data sources are of interest to a variety of stakeholders, including governments, financial institutions, NGOs, and litigation bodies^22–25^. Governments use this information to regulate the industry and monitor compliance with environmental regulations. Financial institutions use the data to assess the risk of investing in the industry and to assess the environmental impact of their investments. NGOs use the data to raise awareness and advocate for more sustainable practices in the industry. Litigation bodies use the data to support legal action against companies that breach environmental regulations.

In order to efficiently decarbonize and transition the cement industry requires robust data and information to support informed decision making and target setting. Some key data needs have already been identified and include: (**1**) production data (this information is crucial to understand the industry’s carbon footprint, track progress towards decarbonization, and measure the impact of decarbonization strategies); (**2**) raw materials sourcing data (important in optimizing production processes, reducing emissions, and promoting sustainable sourcing practices); (**3**) energy sources mix (as the energy intensity of cement production is a key determinant of emissions, accurate data on energy consumption, sources, and efficiency can help identify opportunities for improvement); (**4**) emissions data (greenhouse gas emissions, including CO2); (**5**) market and demand data (market trends and consumer preferences can help identify opportunities for sustainable cement products and drive demand for low-carbon solutions); (**6**) techno-innovation data (uptake of emerging low-carbon technologies and innovations, as well as their feasibility, cost, and impact). In addition, ensuring data transparency, accuracy, and accessibility is crucial in fostering trust and collaboration across the industry, policymakers, and stakeholders towards a common goal of decarbonizing the cement sector.

Literature sources^2,3,5,6,10,13,21,26^ identify several reasons for discrepancies between global cement production capacity and actual production outputs across various regions, specifically due to external (uncertainties of the market demand and competition, sector specific and broader regulations, infrastructure disruptions, energy costs, etc.) and internal (operational issues and internal finances) to the companies factors. Among those, raw material shortages (the availability of limestone, the primary raw material for cement production, can influence production levels) and age of facilities (older facilities might not be as efficient or might face more frequent breakdowns, leading to lower actual production compared to newer ones) have been identified as some of the most available variables for connecting role of internal and internal factors to the utilisation rates of production plants^10,21,26^.

Some of the information identified above are already available, however, are poorly integrated into a single source of reference for decision-makers. Traditional data sources for cement production data include industry reports, government statistics, and company financial statements. For instance, the U.S. Geological Survey provides data on the production and consumption of cement in the United States, while the World Cement Association (WCA) releases annual reports on global cement production and market trends. Company financial statements also provide valuable information on cement production, such as production capacity and utilization rates. Raw materials data can be obtained from trade organizations, such as the WCA, or government agencies, such as the Department of Energy (DOE). The DOE tracks the import and export of key raw materials, such as limestone and clay, used in cement production. Industry reports, such as those released by market research firms, also provide data on the price and availability of raw materials. Energy data is available from a variety of sources, including the DOE and the International Energy Agency (IEA). The IEA tracks energy consumption and production data for the cement industry globally, while the DOE provides data on energy usage and costs for cement production in the United States. Emissions data is typically obtained from government agencies, such as the Environmental Protection Agency (EPA). The EPA tracks emissions data for cement production facilities, including emissions of greenhouse gases and other air pollutants. The WCA also releases data on emissions and energy usage in the cement industry. Market demand data for the cement industry can be obtained from various sources, including industry reports and market research firms. The WCA releases annual reports on market trends and demand, while market research firms such as S&P Global provide data on market demand and pricing trends for cement and concrete products. Additionally, company financial statements and analyst reports provide insight into market demand for specific cement companies and their products.

In this paper we address this issue by developing the first globally comprehensive open sourced asset-level dataset of cement production facilities and their likely supply sources (without ownership identification at this stage). The final database contains detailed facility characteristics including: the exact location of facilities by country, district, city, and latitude and longitude, the immediate company owner and ultimate parent company referenced using open source unique identifiers (including PermID, Legal Entity Identifiers (LEIs) and ticker and exchange information), as well as specific asset-level information including plant type, such as integrated or griding facility, kiln type, such as wet, dry, or semidry kiln, capacity information for both the plant and the kiln, and the year production started (what is not included is information on annual output from each facility), and upstream sourcing attribution (origin of supply (domestic, import), use of raw materials (limestone, sand, iron ore, bauxite), use of clinker in cement production, latitude and longitude of suppliers).

## Methods

### Overview of cement sector

Cement production takes place all around the world, however there is a concentration of production in certain regions of the world. The total cement production in 2020 was estimated to be 4.1 billion metric tons. The top five cement producing countries (China, India, Vietnam, United States and Indonesia) account for approximately 68.2% of global cement production in 2020, with China alone accounting for over 60% of the total global production (4.2 billion metric tons in 2021, according to National Bureau of Statistics of China).

There are several steps involved in the production of cement. Each of these steps provide distinctive characteristics that can be used to identify the production facilities remotely. The first step involves creating a mixture of approximately 90% limestone and other materials, including clay, iron ore and bauxite. Most production takes place close to local limestone quarries to avoid the unnecessary expense associated with transportation. Limestone quarries are typically very large and can easily be visually distinguished from the surrounding environment.

The next step in the production process is the creation of clinker, which is the most energy and emissions intensive step in the cement production process. In clinker production the mixture of raw materials is fed into a kiln heated to over 900 °C, which transforms the limestone (*CaCO*_3_) into lime (*CaO*) and *CO*_2_^16,17^. This is called the calcination process and results in the raw materials reaching temperatures of up to 1450 °C, which results in the formation of clinker. These extreme temperatures provide a distinctive heat signature, which is observable in thermal imagery.

There are several types of kilns that use different production processes, which have large impacts on energy consumption and *CO*_2_ emission intensity. The two main clinker production processes are wet and dry. The key difference between these processes is that in wet production the raw materials that are fed into the kiln are in a slurry form, whereas in a dry process the raw materials are dried out in a pre-heater prior to being put in the kiln. The wet process is a relatively outdated method, which is used less frequently as it is a less efficient process that produces higher emissions and typically creates lower quality cement. As such, it is important to capture the distinction between production processes used at different facilities within an asset-level database. Approximately, 80% of the global clinker production now takes place through a dry process kiln (numbers vary between 60 to 95% in different countries).

Finally, the clinker is ground and mixed with different ingredients to produce cement. The different ingredients that are mixed with the clinker are mostly by-products from other industries such as from blast furnaces, or fly ash from coal power plants^18^. This grinding process is often undertaken at integrated production facilities where the clinker is produced. However, there are also independent grinding facilities. Clinker is transported to these grinding facilities where the final cement mixture is produced and is used to serve the local community. These grinding facilities are typically much smaller in size than the integrated facilities. The cement grinding process accounts for approximately 10% of the total emissions associated with the cement industry, primarily stemming from energy requirements^19^. To fully spatially capture global cement production assets it is important to identify and characterise both the integrated facilities as well as the independent grinding facilities.

### Identification and characterisation of cement plants

**Fig. 1:**
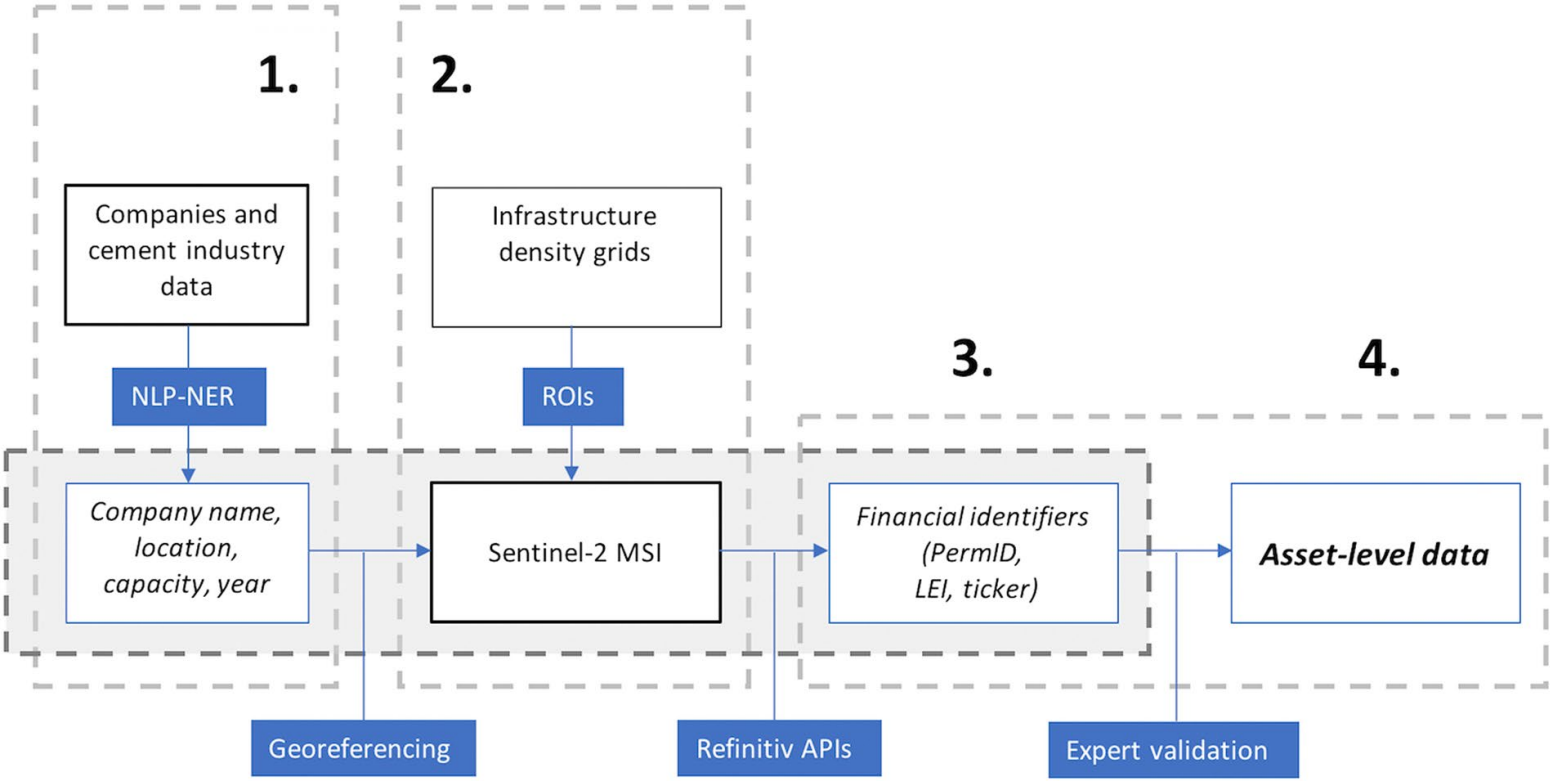
Overview of the methodological workflow: (1) Scraping and collating relevant text resources, containing asset-level details and characterisation of the plants, using publicly available information, (2) Development of an automated model based on Sentinel 2 imagery for the macro localisation of new assets, (3) Deployment of authoritative financial APIs in order to attribute ownership and other type of global financial identifiers for publicly listed companies, and (4) Expert validation of the assembled final data product.

We constructed the database using a variety of techniques to identify and characterise relevant cement production facilities (Fig. 1).

Our process for identifying plants involved (1) scraping and collating relevant text resources, containing asset-level details and characterisation of the plants, using publicly available information, (2) developing an automated model based on Sentinel 2 imagery for the macro localisation of new assets, (3) deployment of authoritative financial APIs in order to attribute ownership and other type of global financial identifiers for publicly listed companies, and (4) expert validation of the assembled final data product.

#### Web scraping & summarisation

Web scraping is a process of collecting data from websites using automated tools. In case of the cement assets application, web scraping was used to collect various types of data that were suggested during the expert-led workshops and deemed essential for monitoring the performance of the industry. To collect cement production data, we used automated tools to scrape websites of cement manufacturers, trade associations, and government and news agencies to retrieve information on the quantity of cement produced by various facilities, their respective capacities (along with associated year of production), ownership references (where available), approximate locations and sourcing of the input materials. The data compiled was therefore designed in such a way that it can be used to track the overall production trends in the industry, identify leading players, and measure the impact of regulatory policies.

As main data collection technology in this study, web scraping is the process of automatically extracting data from websites, which usually involves sending HTTP requests to the website’s server, receiving the HTML source code in return, and parsing the code to extract the desired information. The scraped data can be stored in a database, spreadsheet, or file for further analysis and use. Web scraping can be also done manually and stored in the similar file formats as some websites have anti-scraping measures in place to prevent excessive scraping. We followed the complete guidelines concerning ethical and legal aspects of data scraping, commonly adopted for data collection from unstructured web resources. Since the quality and accuracy of the scraped data can vary, we subsequently validated the entries using geospatial, natural language and manual procedures.

#### Using Large Language Models for extraction of named entities

Large language models (LLMs) achieved a remarkable performance in the recent years across multiple classification and summarisation tasks, including recognition of static linguistic entities (such as proper names, geographic locations, quantities and dates). In our work with the scraped unstructured text data, we extracted such entities of interest as company names, production capacities, years company started operating and their locations (where available), using a combination of deep learning techniques and named entity recognition (NER) algorithms. The process typically starts with the model being trained on a large dataset of labeled text, where the named entities have been tagged in advance. During training, the model learns to identify patterns and relationships between words and phrases that are indicative of named entities, such as proper nouns, organizations, locations, etc. Once trained, the model can then be applied to new text, where it uses its learned knowledge to identify named entities. This is typically done by first preprocessing the text to separate out individual words and tokens, and then passing each token through the transformer model. The model uses a combination of attention mechanisms and fully connected layers to process the tokens and make predictions about which tokens represent named entities.

The output of the transformer model is then processed by NER algorithms, which use additional rules and heuristics to further refine the predictions and assign named entity tags to the tokens. This can include checks for context and grammar, as well as additional information from external sources, such as gazetteers and ontologies.

In this work we used integrated spacy-transformers package, which provided spaCy model pipelines that wrap Hugging Face’s transformers package, resulting in convenient access to state-of-the-art transformer architectures, such as BERT, GPT-2, XLNet and many others. The NER entities, such as Year, Facility and Parent company were directly used in the new database as the candidate entries, while Location was subsequently georeferenced with Google Places API for further verification on the subject of coordinates’ precision.

#### Sentinel-2 RGB model

Next, we used the EarthAI platform to build a Sentinel-2 RGB for exact identification of the cement assets as opposed to the false positives identified by the spatial macrolocalisation model. Using Sentinel-2 Level-2A (L2A) images, available from the EarthAI catalog, we created training and validation image chips that are 300 × 300 pixels in size (about 3 km × 3 km), where the three layers correspond to the red, green, and blue bands (respectively). Sentinel-2 has a high revisit rate (3–5 days), and we do not expect the visual bands to show strong seasonal variance for these industrial targets, except for possible snow coverage in the winter months. We were therefore able to constrain our study to use only high-quality imagery (with scene cloud coverage <5%) captured between May 1, 2020 and August 31, 2020.

In the initial implementation of the Sentinel-2 model, the training/validation chips had the cement plants located at the centre of the chips. While this produced excellent recall on the validation set, we discovered that on deployment, the actual recall achieved for chips intersecting with known cement plants was very low. This was because the chipping scheme did not accurately reflect the situation we faced on model deployment, where the geographical region to score is divided into a regular grid of 300 × 300 pixel tiles, and it is not guaranteed (or even probable) that the cement plants will be centrally located within the tiles.

To create a more robust training/validation data set that better addresses the requirements of the deployment needs, we created chips by offsetting the chip centre from the plant centre by generating a random number drawn from a uniform distribution between 0 and 150 pixels, or half the chip size (in each dimension). An added benefit to this approach is that we can actually increase the size of our training/validation samples by sampling each plant more than once, with a different random offset. For each of the 845 cement plants with known locations in China, we created four chips where the plant is located in different quadrants of the chip extents. To create the land cover examples, we generated eight image chips that surround, but do not intersect with, each plant. In total, we created 3,383, and 11,099 Sentinel-2 RGB image chips for the cement, and land cover classes (respectively) to use for model training and validation.

Sentinel-2 imagery products are stored as images that are 100 × 100 square kilometers in the UTM/WGS84 projection, arranged in a pre-defined, fixed grid. Since China covers several UTM zones, we score the model separately on each image to avoid performing time-consuming coordinate reference system reprojections. In total, there are 1,094 unique grid extents that cover the full deployment region. For each, we calculated the intersection of the grid extent with the deployment region, and created a regular grid of 300 × 300 pixel tiles so that the chip sizes match those used in model training. This results in a total of 415,036 chips to score across the 3.7 million square km deployment region.

As in model training, we created chips where the first, second, and third layers correspond to Sentinel-2 red, green, and blue bands, respectively, and we scored images captured between May 1, 2020 and August 31, 2020. Given the 3–5 day revisit rate of Sentinel-2, there are several images per chip within the May - August time window. For each chip, we select the image with: (a) the lowest reported scene cloud coverage, and (b) zero NoData values within the chip extent. In order to obtain model scores across the full deployment region, we do not impose the 5% threshold on scene cloud coverage as we did in model training. We find that 85% of the chips scored have scene cloud coverage < = 5%.

Every chip is scored using the cement production facility. Model scores - or probabilities - range from 0 to 1, with higher values indicating a higher likelihood of the chip containing a cement plant. As an initial evaluation of the model deployment, we compared the distribution in model probabilities for the full deployment region (415,036 chips) to that of chips intersecting known cement plants. We find that the mode of the distribution for the full deployment region is 0.2, while the probabilities for chips intersecting known plants are skewed considerably higher. The model achieves a realised recall of 83.6% on deployment, which is slightly lower than the recall estimated from the validation set (89.1%).

#### Ownership, and global financial identification

To identify the owner of a facility we first constructed a list of the largest global cement producers. For each producer we their scraped disclosures to identify specific assets that they own (using NER-NLP method mentioned above). Once an owner of an asset was identified the direct (subsidiary) owner as well as the ultimate parent was noted in the database. In most cases we define the ultimate parent as the majority stakeholder of the plant. However, for joint ventures we make a note of both ultimate parents and their ownership stake. Along with the names of the owner and parent, the source of this information was noted alongside the date the information was obtained. This referencing system is important for the transparency of the database as well as providing a useful method for determining if a change in ownership has occurred.

The owner and ultimate parent names were queried in Refinitiv’s OpenPermID database. The PermID is an open source unique identifier provided by Refinitiv, which is useful for distinguishing between different entities around the world. Once a PermID has been assigned to a parent further details are then also extracted from the PermID database. These details include the Legal Entity Identifier (LEI), whether the entity is publicly traded or not, and the primary ticker and exchange information if the entity is publicly traded. For those entities that are not included in the PermID database, no PermID is provided, which is likely if the owner/parent are state-owned enterprises or are very small privately owned producers. All of this information provides consistency of ownership details and facilitates the incorporation of other data, in particular financial datasets.

#### Sourcing typology and production input materials

During the NLP stage of the analysis we also assembled an additional set of the attributes, containing the descriptors about indicative sourcing routes (i.e., ‘local’, ‘imported’ or ‘hybrid’ (using both locally mined and imported materials) and the types of inputs (i.e., ‘clinker’, ‘coal’, ‘limestone’, ‘sand’, ‘bauxites’, ‘iron ore’, and others (a complete list of extractives is available in the database). We used external database, produced by Maus *et al*.^27^, in order to propose spatial dependency matrix between cement production facilities and their upstream suppliers.

## Data Records

Our data records provide spatial information including the near-approximate (within r = 10 km) location for 3,117 cement production assets. All data records are available for download in excel format from the Dryad Repository^28^.

For each asset we provide detailed location information including exact coordinates (WGS84), city, state, country, three-letter iso code (ISO 3166-1 alpha 3), three digit country code (ISO 3166-1 numeric), sub-region and region. The full location details provided were obtained by reverse geocoding the coordinates using Google’s Geocoding API.

Plant characteristic information is also provided, which includes plant type (integrated or grinding), production type (wet or dry), plant capacity and year production first started. For capacity a source is also provided. For capacity figures that were obtained from reported information a link is provided to this source.

Finally, detailed ownership information is provided. This includes the name of the direct or subsidiary owner and the ultimate parent. For both the direct owner and ultimate parent the PermID is provided, where available. The Legal Entity Identifier (LEI), holding status (public or private), ticker and exchange are also provided for the ultimate parent. In the case of a joint venture the ultimate parent information for both ultimate parents is provided. A link to the source of the ownership information is also included.

**Fig. 2:**
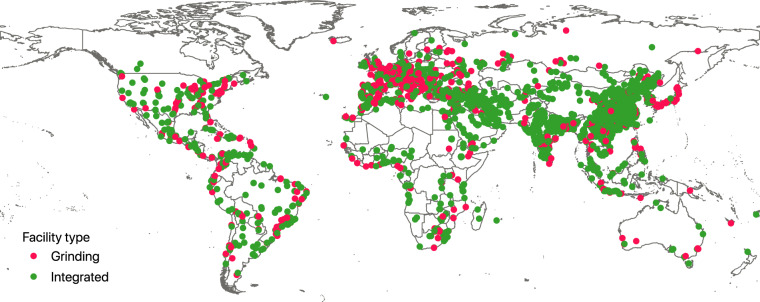
Global distribution of grinding and integrated cement production facilities.

**Fig. 3:**
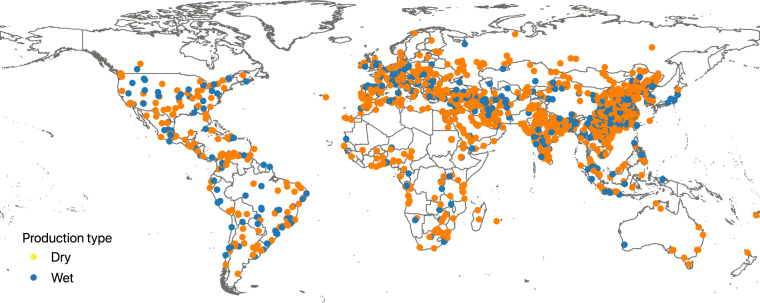
Global distribution of wet and dry cement production technologies of the integrated plants.

**Fig. 4:**
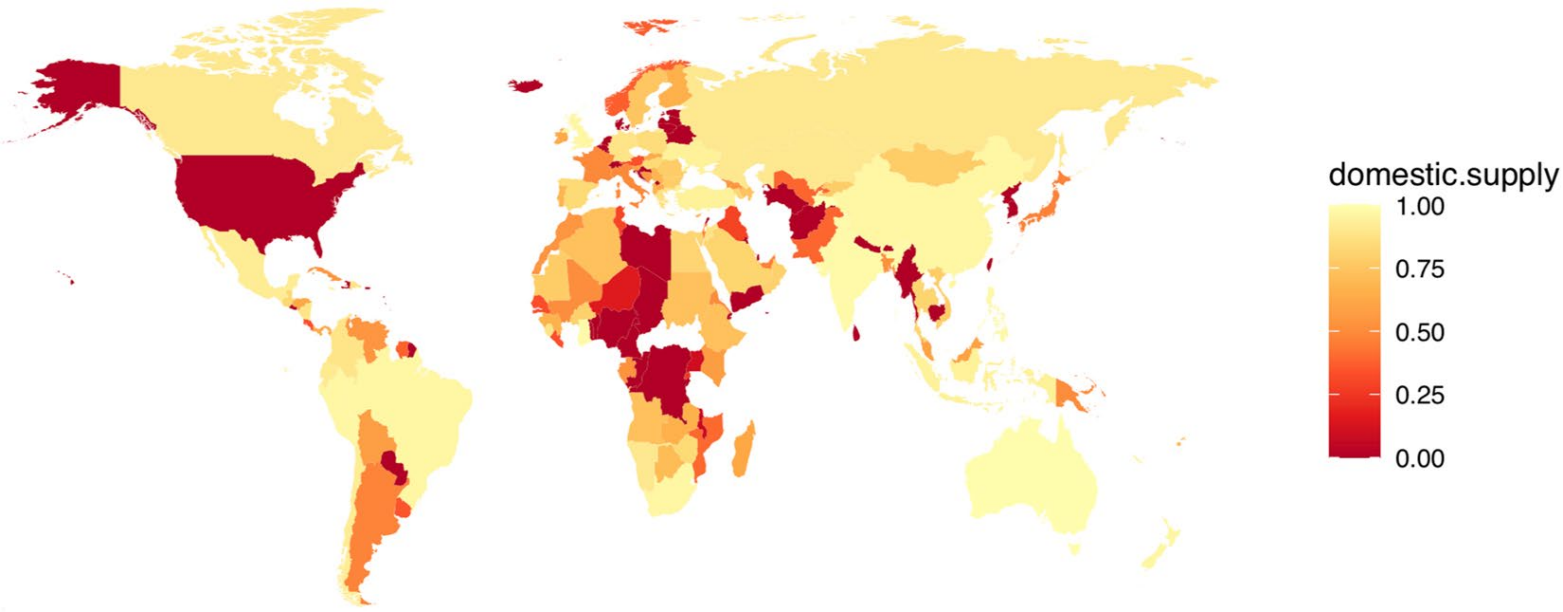
Dependencies of the national cement production facilities on local and imported raw input materials.

Figures 2, 3 show the geographical distribution of the geolocated cement plants. As expected a majority of plants identified are located in China, accounting for 1,159 plants or 37.0% of total assets identified. The next ten countries account for 28.1% (883 assets) of the database. The final 34.9% of assets are spread relatively evenly across 152 other countries. These findings highlight the concentration of production that occurs within the top producing countries and also illustrates the importance of understanding and effectively mapping the sector in these countries. Using sourcing typology and production input materials information, we summarised in Fig. 4 the relative dependencies of various countries on external imports during cement production phases.

A summary of the completeness of the database for each characteristic is provided in Table 1.

**Table 1:** Overview of final database based on the number and the percentage of assets that we have characteristic information for in the final cement database broken down by plant type.

Type	No. of Facilities						
	Count	Ownership	Capacity	Year Start	Sourcing	Raw/m	Clinker
Integr.	2,473 (79%)	1,668 (67.6%)	6,353.7 (100%)	760 (30.7%)	2,466 (99.7%)	977(39.5%)	171 (6.2%)
Grind.	644 (21%)	508 (79%)	1,132.7 (100%)	270 (41.9%)	642(99.7%)	215(33.4%)	69 (10.7%)
Total	3,117 (100%)	2,176 (69.8%)	7,486.4 (100%)	1,030 (33.0%)	3,108 (99.7%)	1,192(38.2%)	240 (8.9%)

## Technical Validation

Throughout the process we have attempted to minimise the potential for these errors to adversely impact the final database. While developing the methodology we consulted with experts in the field to better understand the complexity of the sector. This expert knowledge proved vital to improve our understanding of the structure of cement plants and how they can be identified and characterised. This served as the basis for the majority of the methodological development and for establishing the processing workflow. The final validation and verification undertaken within the team was done in an effort to limit the potential impact of missing some cement assets as well as reducing the possibility of incorrectly identifying a cement plant.

### Comparing asset-level production capacity with the actual production outputs

Utilisation rates are asset-level attribute, conditioned by multiple internal and external factors and which is not consistently reported^21^. Aiming to determine the completeness of the database compared to reported production figures, we identified three possible approaches (**a**) identification of the median utilisation trends for each country or region and compare them against actual production figures (aggregated modelling approach); (**b**) by means of remote sensing tailored for continuous monitoring of high resolution production facilities (Earth Observation approach). Both directions are currently extensively covered by the grey literature sources, and we propose to evaluate them as part of the future research works. Finally, there is also a (**c**) multivariate modelling approach, which allows to make use of various industry and market trends which can determine utilisation trends of individual or groups of cement production facilities.

As a first step towards understanding the completeness and validity of our dataset we have conducted a comparison of the database with reported production and capacity figures. As highlighted in Table 1, we obtained potentially complete information on production capacity for all plants, which were assembled together using mixed media methodology.

Discrepancies between production capacity of the assets and their actual production outputs are known to exist across all manufacturing sectors, not just cement manufacturing. However, given the capital-intensive nature of cement production and its ties to the broader economy (due to construction infrastructure projects demands), the cement industry may be particularly sensitive to some of these factors. A comparison of estimated and reported figures is reported in Table 2. The estimated production using this method provides relatively consistent values compared to the USGS reported production in 2021.

As a recommendation for future work in this direction we identify the application of Earth observation techniques as a groundbreaking avenue for monitoring the activities of cement production facilities. By harnessing the capabilities of satellite-based sensors, it will be beneficial to explore detection and estimation of particulate aerosols emitted during cement production, providing a real-time measure of environmental impact. Furthermore, changes in site temperature can be tracked to understand energy utilization and monitor equipment operations. Implementing Earth observation methodologies will not only elevate the accuracy and timeliness of data collection but also can underscore a commitment to environmental responsibility and sustainable industrial practices. Although it could be costly, we nevertheless recommend the adoption of these techniques for a holistic and efficient monitoring framework for cement production facilities.

**Table 2:** Updated production capacity of assets (H1/2023) vs. reported production by region in 2019 from the USGS (without accounting for utilisation rates of production facilities).

Region	Cap/Rep(millions tons)	Reported Production (2021) (millions tons)	Production Capacity (2023)(millions tons)
Asia	5,970.4	3,268.0	+ 45%
Africa	361.7	212.5	+ 41%
Europe	638.4	253.5	+ 60%
Americas	488.9	276.9	+ 43%
Oceania	26.9	12.0	+ 56%
Global	7,486.4	4,132.2	+ 45%

## Usage Notes

The primary data format is the CSV (comma-separated values), which can be read by any well-known and widely used commercial (e.g., MS Excel) or open source (e.g., Open Office/Libre Office) software programs. It can also be read into *python* environments by using *pandas* or *geopandas* packages.

## Data Availability

The deployment phase code notebooks are available from the GitHub Spatial Finance repository: [1] Spatial macrolocalisation model; [2] Cement production types and capacity estimation models; [3] Sentinel-2 cement assets deployment models.
